# A pilot study of alterations of the gut microbiome in canine chronic kidney disease

**DOI:** 10.3389/fvets.2023.1241215

**Published:** 2023-08-24

**Authors:** Kyung-Ryung Kim, Seon-Myung Kim, Jung-Hyun Kim

**Affiliations:** ^1^Department of Veterinary Internal Medicine, College of Veterinary Medicine, Konkuk University, Seoul, Republic of Korea; ^2^KR Lab Bio Incorporation, Seoul, Republic of Korea

**Keywords:** dog, chronic kidney disease, gut microbiome, dysbiosis, proteolytic bacteria, saccharolytic bacteria

## Abstract

**Introduction:**

Gut dysbiosis has been noted in humans and animals with chronic kidney disease (CKD). However, little is known about the gut microbiome in canine patients with CKD. This study aimed to analyze and compare the gut microbiome profiles of healthy and CKD dogs, including differences in the gut microbiome between each CKD stage.

**Methods:**

The study was conducted on 29 client-owned dogs who underwent physical examination, complete blood count (CBC), serum biochemistry, and urinalysis. The gut microbiome profile of healthy dogs (*n* = 10) and dogs with CKD (*n* = 19) was analyzed employing 16S rRNA sequencing.

**Results:**

Significant differences were seen in the composition of the gut microbiome, with increased operational taxonomic units from the phylum *Proteobacteria* (*p* = 0.035), family *Enterobacteriaceae* (*p* < 0.001), and genus *Enterococcus* (*p* = 0.002) in dogs with CKD, and a decrease in the genus *Ruminococcus* (*p* = 0.007). Furthermore, an increase in both the progression of CKD and abundance of genus *Klebsiella* (Jonckheere-Terpstra test statistic value (JT) = 2.852, *p* = 0.004) and *Clostridium* (JT = 2.018, *p* = 0.044) was observed.

**Discussion:**

Our study demonstrated that in dogs with CKD, the composition of the gut microbiome varied depending on the stage of CKD. Alterations in gut microbiome composition observed in CKD patients are characterized by an increase in proteolytic bacteria and a decrease in saccharolytic bacteria. These findings suggest specific gut microbiota could be targeted for clinical management of uremic dogs with CKD.

## 1. Introduction

In dogs, chronic kidney disease (CKD) is the most prevalent kidney disease, accounting for up to 7% of the cases (1). Dogs with CKD experience similar manifestations as humans, in that both are characterized by progressive functional or structural changes in one or both kidneys (2). Initiated by numerous factors, including familial, congenital, and acquired properties, CKD results in a reduced total kidney glomerular filtration rate and may ultimately lead to uremic crises and death (3).

The intimate relationship between uremic diseases, including CKD and gut microbiome profiles is an emerging topic of research in the field of human and veterinary medicine (4, 5). In human medicine, pathogenic links between the gut microbiome and CKD progression have been described, including increased gut permeability and excessive production of microbiota-generated nephrotoxin (6, 7). Additionally, in cats and human patients with CKD, abnormal gut microbiome composition has been reported (8–10). Dysbiotic intestinal microbiome and CKD promote each other through various pathophysiological mechanisms (11). In human medicine, patients with CKD can be identified by an inequality in the types of microbiomes, with a higher prevalence of proteolytic microbiomes over saccharolytic ones (12). This imbalance accelerates the progression of CKD, since saccharolytic bacteria provide protection against inflammation associated with CKD and help prevent the progression of the disease (12, 13).

Recently, the concept of “enteric dialysis” in patients with CKD has attracted much attention, especially, regarding the need to consider intestinal health when devising treatment strategies for CKD (11). For devising novel therapeutic approaches targeting gut microbial communities to alleviate the progression of CKD, the analysis of the gut microbiome of healthy dogs and of those with CKD is essential. However, the microbiome characteristics of dogs with CKD have not been well studied yet. In this study, we sought to characterize the gut microbiota in dogs with CKD compared to healthy dogs and explore potential relationships between gut microbiota composition and CKD at different stages.

## 2. Materials and methods

### 2.1. Study design and selection of animals

This prospective study was performed at Konkuk University Veterinary Medical Teaching Hospital between March and December 2022. All protocols of this study were reviewed and approved by the Konkuk University Institutional Animal Care and Use Committee (approval number: KU22036-1). Each owner provided informed consent for their dog to participate in the study. Healthy dogs were enrolled based on their history, physical examination, complete blood count, and serum biochemistry. Dogs were excluded from the healthy group if they were diagnosed with gastrointestinal disease, cancer, liver disease, CKD, or urinary tract infection.

Dogs were considered to have CKD if either of the following criteria were fulfilled: (1) increased serum concentration (Cr ≥1.6 mg/dL) on at least two occasions or (2) increased serum symmetric dimethylarginine (SDMA >14 μg/dL) on at least two occasions. CKD was staged according to the International Renal Interest Society (IRIS) guidelines. Exclusion criteria were the administration of antibiotics or probiotics within 4 weeks before sample collection, pregnant females, and dogs with other abnormal clinical findings, including malignant tumors, urinary tract infections, or gastrointestinal diseases. All dogs that participated in the study were fed commercial or prescription diets with no regulation of ingredient composition.

In total, nineteen dogs with CKD (IRIS stage 1, *n* = 5; stage 2, *n* = 6; stage 3, *n* = 4; and stage 4, *n* = 4) and ten healthy control dogs were included in this study. The gut microbiome was analyzed for all dogs to examine and compare its structure in the CKD and healthy groups.

## 3. Microbiome sample collection

Rectal swab samples were collected from healthy and CKD dogs using a sterile culture swab applicator (REST™ NBgene-GUT, Noble Bio, Hwaseong, Korea). The swabs were stored at 4°C until further analysis.

### 3.1. DNA extraction and 16S rRNA sequencing

Bacterial total genomic DNA was extracted using a PureLink™ Microbiome DNA Purification Kit (Invitrogen, Waltham, MA, United States) according to the manufacturer’s instructions. Each DNA sample was eluted in 50 μL of S6 Elution buffer. The concentration and purity of the eluted DNA were assessed using a NanoDrop ND-1000 spectrophotometer (Thermo Fisher Scientific, Wilmington, DE, United States) and Qubit (Thermo Fisher Scientific).

DNA was extracted from stool samples and used as a template for PCR amplification of the V3–V4 variable region of the bacterial 16S rRNA gene using barcoded primers containing adaptors for the Ion S5™ sequencing system (Thermo Fisher Scientific). Each reaction mix contained Platinum PCR SuperMix High Fidelity (23 μL), 10 μM F Primer (1 μL), 10 μM R Primer (1 μL), and 2.5 ng/μL genomic DNA template (2 μL). Thermocycling conditions for PCR amplification were as follows: 3 min at 94°C, followed by 25 cycles of 30 s at 94°C, 30 s at 50°C, and 30 s at 72°C, a 5 min final extension at 72°C, and hold at 4°C. PCR products were purified using AMPure™ XP reagent according to the manufacturer’s instructions. The amplicons were quantified using a Qubit dsDNA HS Assay Kit (Invitrogen, CA, United States, Q32854). After quantification, each amplicon was diluted to a concentration of 100 pM. Equal amounts of diluted amplicons were collected in 1.5 mL tubes to make a final volume of 50 μL.

### 3.2. Bioinformatics analysis of the sequences

Sequencing data were analyzed using Quantitative Insights into Microbial Ecology (QIIME) in the Thermo Fisher Ion Reporter Software v5.18.4.0. The default options included a read length filter ≥150, minimum alignment coverage ≤90.0, genus cutoff ≤97.0, species cutoff ≤99.0, and slash ID reporting percentage ≤ 0.2. The read abundance filter was set at ≤5, which is not the default value. Sequences were clustered at the operational taxonomic unit (OTU) level using Greengenes v13.5, curated microseq(R) 16S reference library v2013.1, and QIIME in the two databases.

To assess the diversity of bacterial species in fecal samples, alpha rarefaction curves were generated using the observed OTUs and Chao1, Shannon, and Simpson diversity indices, and OTU metrics were identified. We then compared the bacterial communities in healthy dogs and dogs with CKD stages 1 through 4 by grouping them together. We used the Analysis of Similarities (ANOSIM) in the scikit-bio (v0.5.8) package in Python (v3.9.7) to assess beta diversity using the UniFrac distance matrix and visualized it using a Principal Coordinate Analysis (PCoA) plot. UniFrac is a phylogeny-based beta diversity metric that calculates the similarity in microbial communities based on the evolutionary distance between microbial lineages. Weighted and unweighted UniFrac were used, with the latter only considering the presence of OTUs in the two groups being compared, and the former also considering the abundance of taxa within the groups. OTU abundance refers to the number of reads or sequences assigned to a particular OTU in the microbial community sample.

In addition, we analyzed the composition of microbiomes (ANCOM) using the scikit-bio package in Python to identify differentially enriched features in the microbiome data. ANCOM uses the W-statistic to test for significant differences in the relative abundance of features between groups of samples, such as healthy control dogs and experimental dogs with CKD. ANOSIM generates an *R*-value between −1 and 1, with values close to 1 indicating a large difference between groups and values close to 0 indicating no significant difference in composition between groups. The W-statistic was used to test for differences in the relative abundance of features between different sample groups, and a number of significantly different features were obtained after correcting for multiple comparisons within the significance level.

### 3.3. Statistical analysis

Statistical analysis was performed using SPSS (IBM Corporation, Armonk, NY). Normality of data was assessed using the Shapiro–Wilk test. Normally distributed data are presented as means (± s.d.), whereas data that did not meet the assumptions of normal distribution are represented as medians and intervals. Continuous variables of the healthy and CKD groups were analyzed using the Mann–Whitney test and independent sample *t*-test for two groups, and the Kruskal–Wallis test was used to compare more than two groups. Post-hoc Bonferroni correction was performed to determine the differences in bacterial taxa between the groups. The Jonckheere–Terpstra test was used to evaluate the significance of trends in the taxa analysis of each CKD and control group. GraphPad Prism 7 (GraphPad Software, San Diego, CA, United States) was used to produce graphs. For all analyses, statistical significance was set at *p* < 0.05.

## 4. Results

### 4.1. Animals

Baseline data, including age, gender, breeds, clinical scores, systolic blood pressure, and hematologic and biochemical properties, are presented in Table 1. As expected, dogs with CKD had significantly increased serum concentrations of blood urea nitrogen (BUN; *p* = 0.003), creatinine (*p* = 0.003), and SDMA (*p* < 0.001). In addition, dogs with CKD had a significantly higher urine creatinine protein ratio (UPCR) than healthy dogs (*p* = 0.009). No significant differences were found in age, gender, fecal score, or systolic blood pressure between the healthy and CKD dogs.

**Table 1:** Demographic, serum chemistries, and urine analytes in healthy (*n* = 10) and CKD dogs (*n* = 19).

Variables (reference interval)	Healthy dogs (*n* = 10)	CKD dogs				*p*-value
		IRIS stage 1 (*n* = 5)	IRIS stage 2 (*n* = 6)	IRIS stage 3 (*n* = 4)	IRIS stage 4 (*n* = 4)	
Age (years)	9 (2–18)	10.5 (2–16)	12 (1–16)	12.5 (11–15)	12 (5–15)	0.772
Gender						
Intact female						
Neutered female	4	3	6	2	1	
Intact male				1	1	
Neutered male	6	2		1	2	
Breeds						
Maltese	2	2	1	2	2	
Poodle	3		3			
Pomeranian	1	1				
Mixed	2		1		1	
Others	2	2	1	2	1	
Fecal score (1–7)	2 (2–3)	2 (2–3)	3 (2–4)	3 (3–4)	3 (2–3)	
Systolic blood pressure (mmHg)	136.5 (125–145)	139 (118–160)	137.5 (110–145)	137.5 (129–163)	143 (119–160)	0.312
Serum BUN (7–27 mg/dL)	14.5 (8–24)^a^	25 (11–25)^b^	24 (15–54)^b^	42.5 (27–72)^ab^	83.5 (54–106)^ab^	*0.003
Serum creatinine (0.5–1.8 mg/dL)	0.65 (0.3–1.1)^a^	0.8 (0.7–1)^b^	1.2 (0.8–1.6)^b^	1.25 (1–1.6)^ab^	6.85 (1.1–24)^ab^	*0.003
SDMA (0–14 μg/dL)	11.5 (7–14)^a^	15.5 (15–17)^b^	26 (19–30)^b^	47 (29–53)^ab^	84.5 (48–100)^ab^	*<0.001
UPCR						
<0.2	9	4	3	3		
0.2–0.5	1	1	1			
>0.5		1	2	1	4	

Variables are expressed as medians and intervals as they were not normally distributed. *p < 0.05, statistically significant differences based on the Kruskal-Wallis test. Different superscript literals (a, b) signify statistically significant difference between the groups. CKD, chronic kidney disease; IRIS, international renal interest society; BUN, blood urine nitrogen; SDMA, symmetric dimethylarginine; UPCR, urine protein creatinine ratio.

### 4.2. Gut microbiome analysis

**Figure 1:**
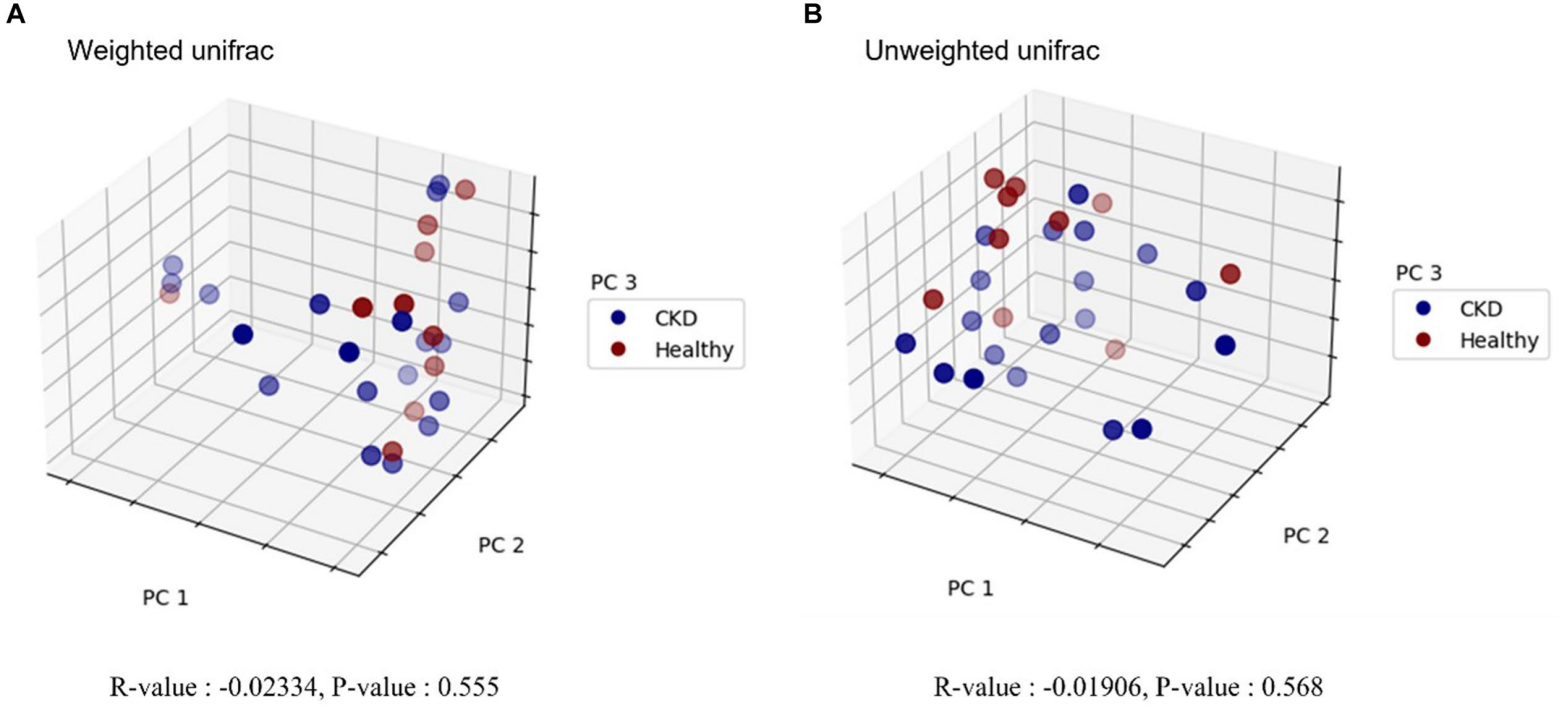
Principal coordinate analysis (PCoA) plot in microbiome of healthy dogs and dogs with chronic kidney disease. Beta diversity analysis using weighted **(A)** and unweighted **(B)** UniFrac distances is shown, respectively. Blue and red circles represent gut microbiome samples from CKD and healthy dogs. Each axis represents the principal coordinate that explains the largest data change, and the spatial distance of respective sample points indicates the similarity between samples. *R*-values and *p*-values of analysis of similarities (ANOSIM) are provided below each graph. *R*-value less than zero indicates that the within-group variability is greater than the between-group variability. No statistically significant difference was detected between healthy and CKD dogs.

Overall, 779 OTUs were found in all the analyzed samples (*n* = 29). Sequence analysis generated 12,738,717 reads from 29 samples. For the evaluation of alpha diversity, Chao1, Shannon, and Simpson indices were compared between healthy dogs and dogs with CKD (Table 2). No clear difference in alpha diversity was observed between the healthy and CKD groups using the OTU, Chao1, Shannon, or Simpson diversity indices. Beta diversity, which was presented with PCoA plots (Figure 1), showed no significant difference in the gut microbiome between healthy and CKD dogs based on ANOSIM of weighted (*R* = −0.02) and unweighted (*R* = −0.02) UniFrac distances. For additional analysis of the differential abundance of OTUs between experimental control groups, ANCOM and ALDEx2 methods were employed; however, neither of the analyses showed a significant difference in diversity between the two groups.

**Table 2:** Alpha diversity results analyzed using 16S rRNA sequencing.

Alpha diversity	Healthy dogs (*n* = 10)	CKD (*n* = 19)	*p*-value
OTU	26.1 ± 8.2	27.3 ± 8.8	0.773
Chao1	15.4 ± 7.2	20.7 ± 8.0	0.090
Simpson index	0.8 ± 0.2	0.7 ± 0.2	0.415
Shannon index	2.0 ± 0.6	1.8 ± 0.5	0.381

Variables are expressed as mean (±SD). SD, standard deviation; OTU, operational taxonomic unit; CKD, chronic kidney disease.

**Figure 2:**
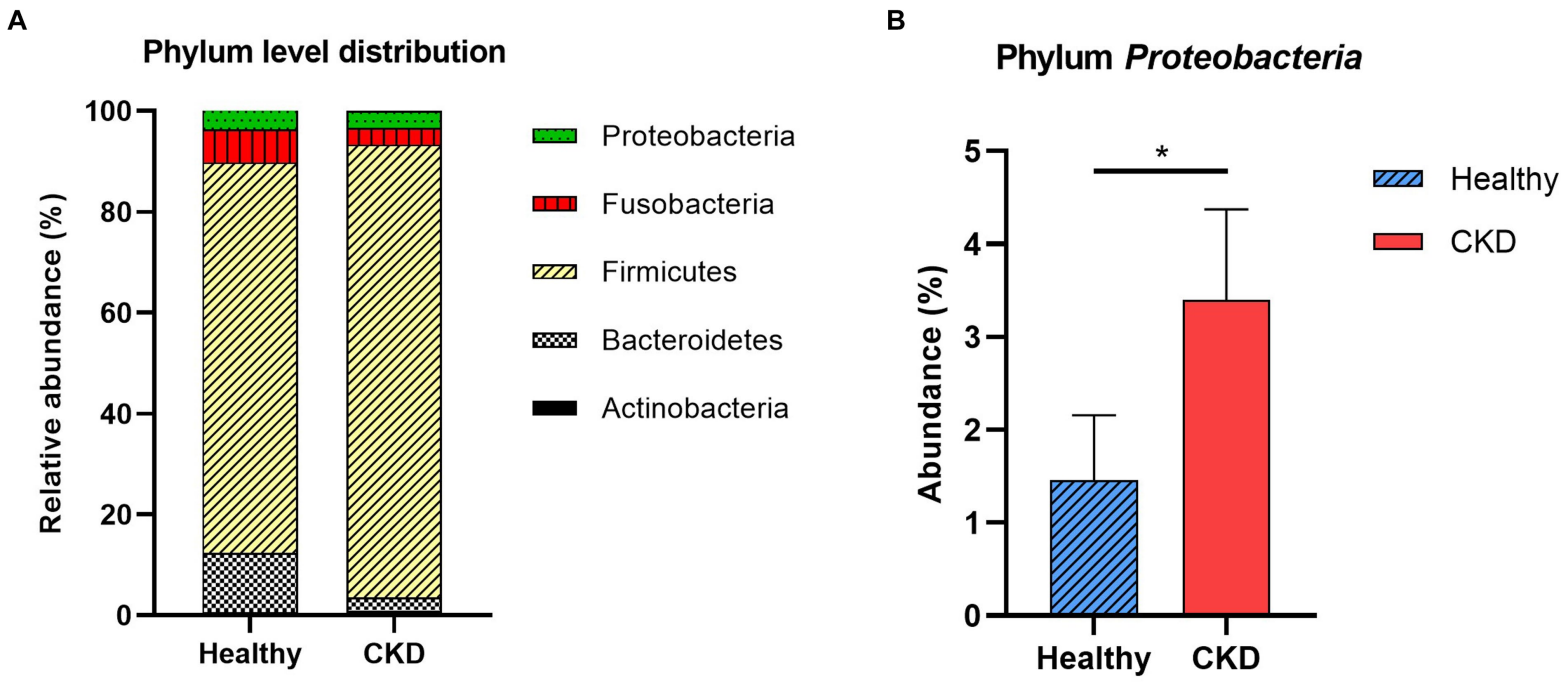
Comparison of taxa analysis of healthy and chronic kidney disease (CKD) dogs at phylum level **(A)** and relative abundance of phylum *Proteobacteria* **(B)**. Mean relative abundance of gut microbiome was shown as stacked bar graph **(A)**. *Firmicutes* accounts for the majority of gut microbiome in both healthy and CKD groups. Mean relative abundance of phylum *Proteobacteria* was shown as bar graph with ± standard error of the mean (SEM; b). The relative abundance of *Proteobacteria* presented notable increase in dog with CKD. *p < 0.05; **p < 0.01; ***p < 0.005; ****p < 0.001.

At the taxonomic level, the following five main phyla were observed: *Actinobacteria*, *Bacteroidetes*, *Firmicutes*, *Fusobacteria*, and *Proteobacteria* (Figure 2). Among these, the most predominant phylum in healthy and CKD dogs was *Firmicutes*, which accounted for more than 85% of the total microbiome. The relative abundance of *Proteobacteria* was significantly higher in dogs with CKD than that in the healthy control group (*p* = 0.035; Figure 2; Table 3).

**Table 3:** Taxa analysis of healthy and CKD dogs at phylum level.

Phylum	Healthy dogs (*n* = 10)	CKD (*n* = 19)	*p*-value
Actinobacteria (%)	0.1 (0–5.2)	0.3 (0–5.5)	0.211
Bacteroidetes (%)	11.8 (0–61.7)	1.4 (0–26.6)	0.195
Firmicutes (%)	80.5 (21.6–99.9)	92.5 (51.8–99.5)	0.164
Fusobacteria (%)	0.1 (0–39.7)	0 (0–45.1)	0.062
Proteobacteria (%)	0.3 (0–6.6)	2.0 (0.14–18.1)	*0.035

Variables are expressed as medians and intervals. *p < 0.05, statistically significant differences based on the Mann–Whitney test. CKD, chronic kidney disease.

**Figure 3:**
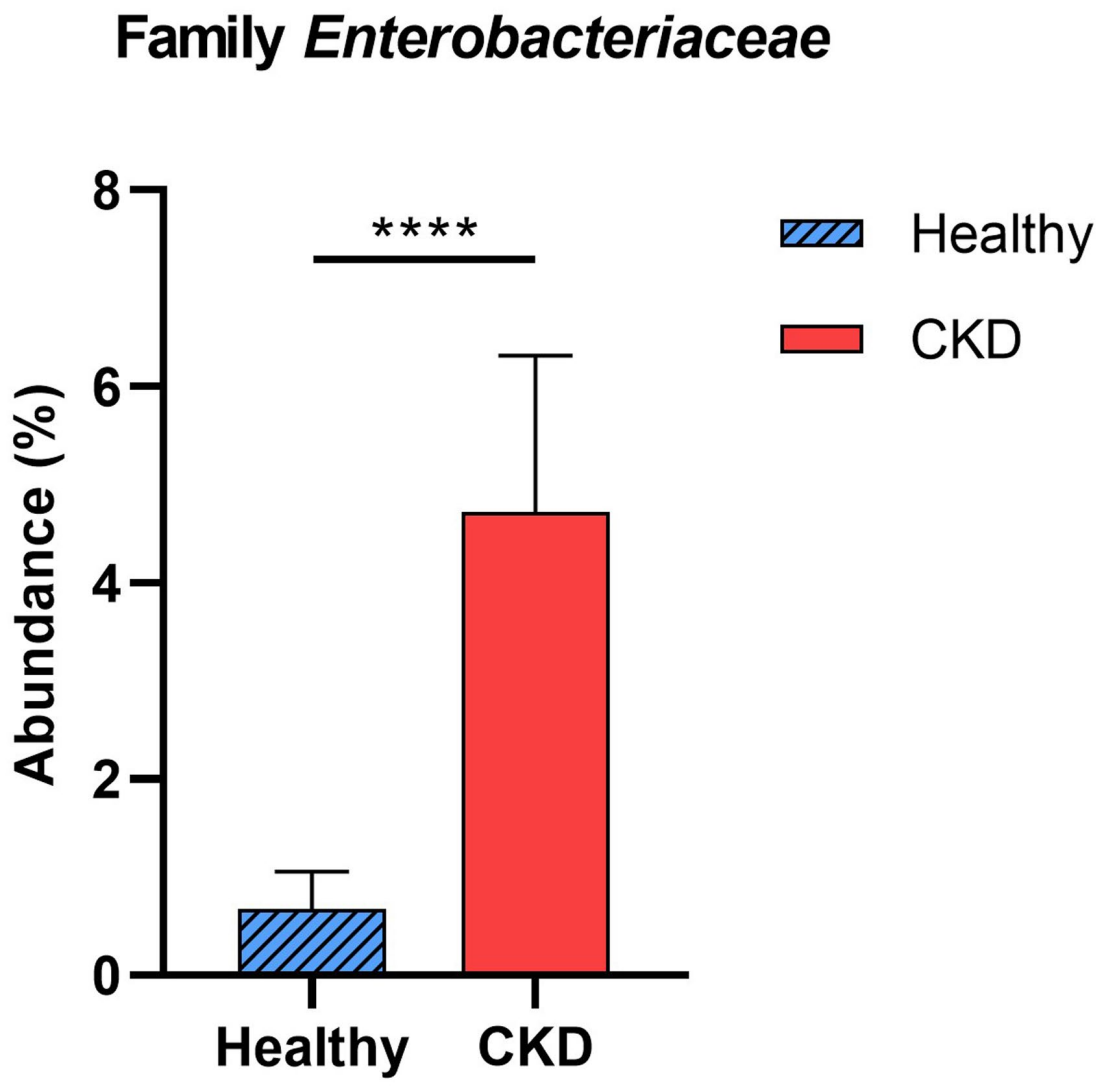
Comparison of taxa analysis of healthy and chronic kidney disease (CKD) dogs at family level. Mean relative abundance of family *Enterobacteriaceae* was shown as bar graph with ± standard error of the mean (SEM). Statistically significant increase in the relative abundance of family *Enterobacteriaceae* was observed in CKD patients compared to healthy dogs. *p < 0.05; **p < 0.01; ***p < 0.005; ****p < 0.001.

*Enterobacteriaceae*, a bacterial family, showed a significant increase in CKD dogs compared with that in healthy control dogs (*p* < 0.001; Figure 3). Other bacterial species producing uremic toxins, including *Bacteroidaceae*, *Pseudomonadaceae*, *Clostridiaceae*, were more abundant in dogs with CKD than in healthy dogs (no statistical significance). No correlation in taxa was found between the microbial communities and CKD progression at the phylum and family levels.

**Figure 4:**
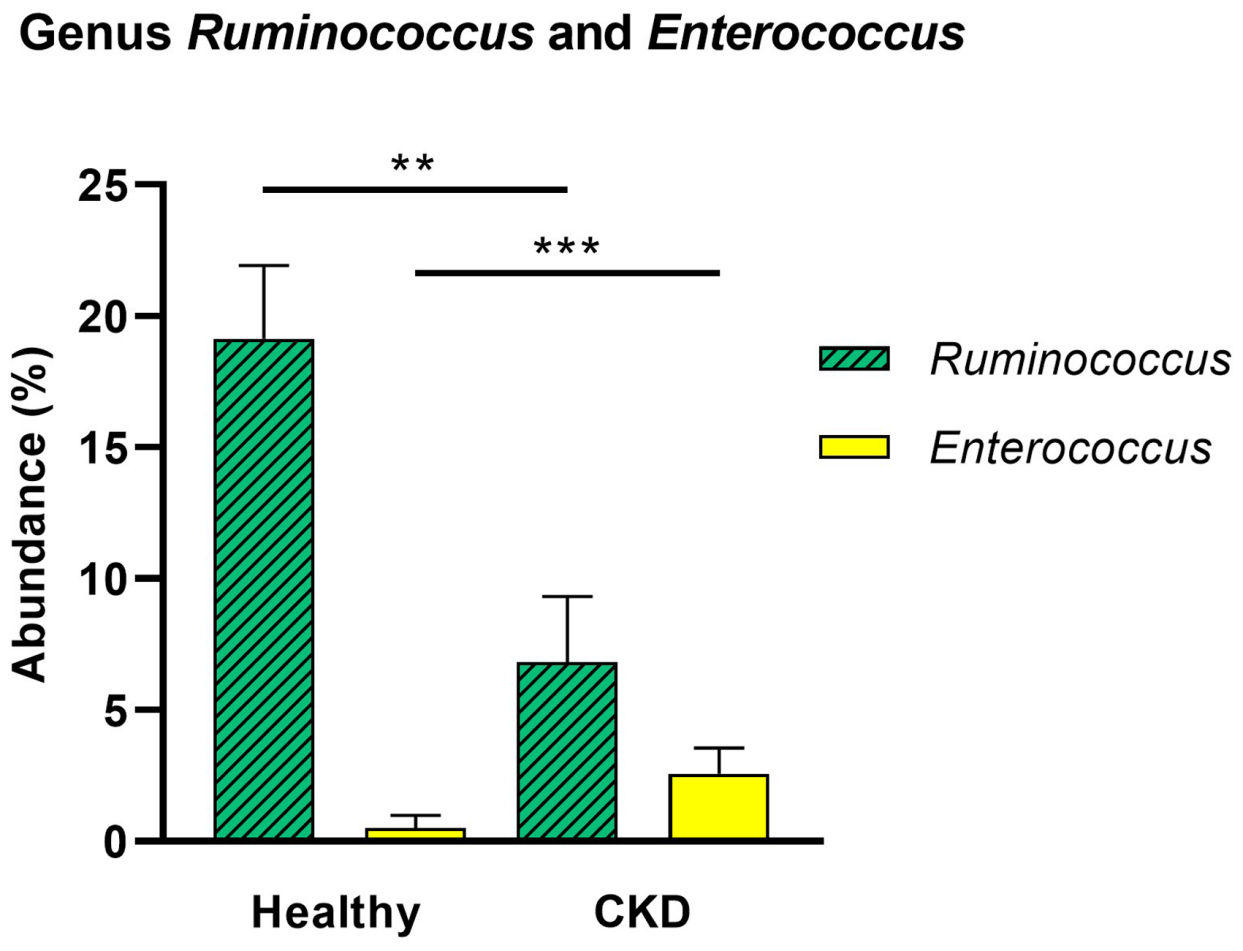
Comparison of taxa analysis of healthy and chronic kidney disease (CKD) dogs at genus level. Mean relative abundance of genus *Ruminococcus* and *Enterococcus* was shown as bar graph with ± standard error of the mean (SEM). Compared to healthy dogs, CKD patients showed statistically significant increase in the relative abundance of genus *Enterococcus* and decrease in genus *Ruminococcus*. *p < 0.05; **p < 0.01; ***p < 0.005; ****p < 0.001.

**Figure 5:**
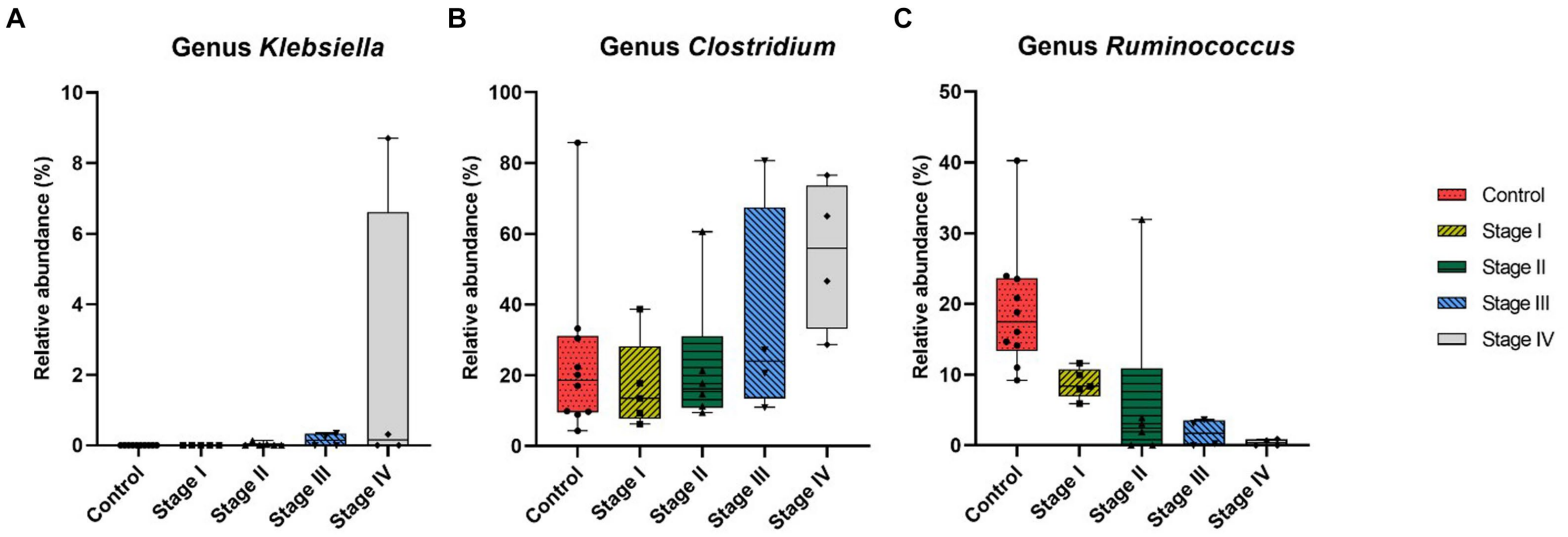
Correlation analysis at genus level of healthy dogs and dogs with chronic kidney disease (CKD) using the Jonckheere–Terpstra test. The relative abundance of genus *Klebsiella* **(A)**, *Clostridium* **(B)**, and *Ruminococcus* **(C)** was measured in healthy dogs and dogs from each stage of CKD. The lower and upper limits of the box indicate the minimum and maximum values, respectively; the line within the box depicts the median values. Each five color indicates control group and 4 stages of CKD. Statistically significant increasing trend in the relative abundance of genus *Klebsiella* and genus *Clostridium* with the progression of CKD was observed, while there was a statistically significant decreasing trend in the relative abundance of genus *Ruminococcus*.

**Figure 6:**
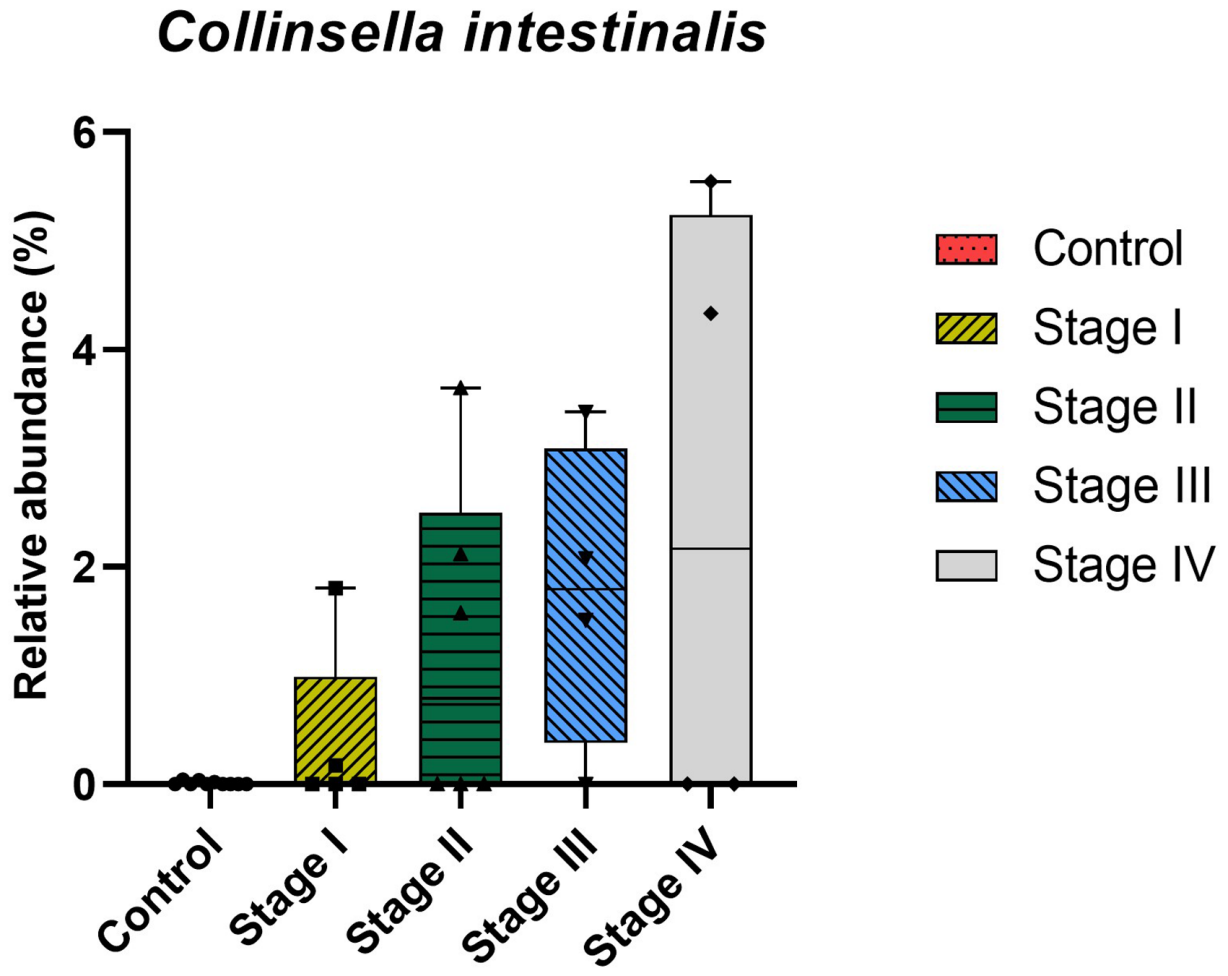
Correlation analysis at species level of healthy dogs and dogs with chronic kidney disease (CKD) using the Jonckheere–Terpstra test. The lower and upper limits of the box indicate the minimum and maximum values, respectively; the line within the box depicts the median values. Each five color indicates control group and 4 stages of CKD. A statistically significant positive trend in the relative abundance of *Collinsella intestinalis* was observed as CKD progressed.

At the genus level, CKD dogs tended to have a significantly higher relative abundance of *Enterococcus* (*p* = 0.002) and lower relative abundance of *Ruminococcus* (*p* = 0.007; Figure 4). Furthermore, increasing trends of CKD progression and relative abundance of the genera *Klebsiella* (Jonckheere-Terpstra test statistic value (JT) = 2.026, *p* = 0.043) and *Clostridium* (JT = 2.018, *p* = 0.044) were observed. In contrast, the relative abundance of *Ruminococcus* was inversely related to CKD progression (JT = −4.311, *p* < 0.001; Figure 5). No significant difference or correlation was found at the species level between dogs with CKD and healthy dogs, except that the relative abundance of *Collinsella intestinalis* increased with the progress of CKD (JT = 2.302, *p* = 0.021; Figure 6).

## 5. Discussion

Recent evidence suggests that gut microbiota dysbiosis plays a critical role in human patients with CKD (14). However, the relationship between altered gut microbiome profiles and CKD remains unclear in dogs. Here, we analyzed the gut microbiome profiles of healthy dogs and dogs with CKD at different stages.

In this study, most predominant bacterial phyla in healthy and CKD dogs were *Actinobacteria*, *Bacteroidetes*, *Firmicutes*, *Fusobacteria*, and *Proteobacteria* which are consistent with findings in the human and veterinary medicine (15, 16). Especially overgrowth of the phylum *Proteobacteria* was observed in dogs with CKD compared with that in the healthy dogs, which is in accordance with previous studies (17, 18). *Proteobacteria*, which is the largest bacterial phylum, is a potential diagnostic marker of dysbiosis. In humans, an increased prevalence of *Proteobacteria* is associated with numerous chronic and inflammatory diseases, including CKD, inflammatory bowel disease, cardiovascular diseases, and chronic lung diseases (14, 19, 20). Although a precise explanation for these phenomena cannot be given, it is generally known that patients with CKD experience increased systemic immune inflammatory responses and intestinal susceptibility.

Hyperproliferation of lipopolysaccharide (LPS) forming bacteria, genus *Klebsiella*, was prominent in patients with CKD in this study. Dysbiosis of the gut microbiome in patients with CKD can cause an increase in systemic inflammation due to the presence of LPS derived from the gut microbiome (21). In addition, a gradual increase in *Collinsella intestinalis* in dogs with CKD was observed in this study; this species belongs to the family *Coriobacteriaceae* and is a pathobiont (22). Some studies have shown that administration of *Collinsella* induces loosening of gut integration by downregulating the expression of tight junction proteins of enterocytes and is strongly related to increased production of proinflammatory cytokines in mouse models. The disintegration of the intestinal barrier is caused by the products resulting from gut microbiome dysbiosis, leading to the translocation of bacterial products (e.g., LPS, uremic toxins, and cytokines) from the gut into the systemic circulation (9, 23). As discussed earlier, the correlation between CKD and gut dysbiosis, intestinal barrier deterioration, gut bacterial translocation, and gut-derived inflammatory molecules suggests a pathophysiological mechanism in the kidney and gut. No clear evidence of systemic inflammation and gut barrier deterioration caused by dysbiosis has been proven in dogs; however, similar findings of dysbiosis in CKD patients and dogs have been observed, which suggests a close underlying renal–gut mechanism.

We also observed that the abundance of families *Enterobacteriaceae*, *Clostridiaceae*, *Pseudomonadaceae*, and *Bacteroidaceae* was higher in dogs with CKD than in the control group. Although no statistical significance was found, except for the family *Enterobacteriaceae*, these bacterial species are well-known to produce uremic toxins (24). At the genus level, *Enterococcus* was significantly more abundant in the gut microbiome of dogs with CKD compared with that in healthy dogs. Comparing each stage of CKD and the healthy group, some proteolytic bacteria, the genera *Klebsiella* and *Clostridium*, showed a gradual increase in abundance as CKD progressed; as both these genera are known to exhibit proteolytic activity, intimate relationship with increased levels of uremic toxins is expected. While individuals with normal kidney function can excrete uremic metabolites, they are retained in CKD individuals, negatively affecting their biological functions (25). The breakdown of aromatic amino acids by proteolytic species due to an imbalance in the gut microbiome has been recognized as a contributor to the production of protein-bound uremic toxins, such as indoxyl sulfate and p-cresol sulfate (26). In humans, the accumulation of toxic amino acid metabolites accelerates the progression of CKD through various pathophysiological mechanisms, including exacerbation of oxidative stress in renal tubular cells, induction of glomerular sclerosis and the accumulation of intracellular toxins (27). These results suggest that gut-derived uremic toxins may contribute to the development and progression of CKD in dogs by synthesizing uremic toxin molecules related to CKD deterioration. Nevertheless, the exact mechanism underlying this phenomenon requires further clarification.

Our study also revealed a significantly decreased abundance of the genus *Ruminococcus* in dogs with CKD, which was responsible for producing short-chain fatty acids (SCFAs). Moreover, we observed a potential association between the abundance of *Ruminococcus* and CKD progression in dogs. *Ruminococcus* spp. are gram-positive anaerobic bacteria that belong to the phylum *Firmicutes*. This genus is a well-known saccharolytic bacteria which produce butyrate that is inversely associated with serum LPS concentrations in humans (28). Butyrate, an SCFA, is mainly produced by saccharolytic bacterial fermentation of dietary fiber in the gut (29). This fatty acid is not only an essential energy source for cells of the lower intestinal tract but is also an immune modulator and blood pressure regulator in humans (30). SCFAs decrease inflammation and suppress oxidative injury and fibrosis of the kidney through numerous biological mechanisms, including through G-protein-coupled receptors, epigenetic regulation, and histone acetylation (29). In human medicine, a reduction in the butyrate-producing bacteria species is associated with CKD progression (9). Although the biochemical function of butyrate in dogs is not well understood, given the significant decline in butyrate-producing bacterial species in dogs with CKD, SCFAs could be considered as a therapeutic target to slow the progression of CKD in dogs. Our results show that dysbiosis in dogs with CKD may promote overgrowth of proteolytic species while reducing the population of saccharolytic species, which may contribute to the accumulation of uremic toxins and a decrease in the production of SCFAs. It also leads to a state of chronic inflammation by translocating bacterial metabolites, cytokines, and inflammatory molecules, which are key therapeutic targets in CKD.

In contrast to human study, our study did not reveal any significant differences in alpha and beta diversity indices between healthy and CKD dogs. This discrepancy might be due to differences in the species of microbial communities. Because many other studies conducted on species other than humans (e.g., rats, dogs) found no significant difference in the diversity of the gut microbiome between healthy and CKD patients (16, 31). Further investigation is required regarding interspecies differences to evaluate changes in the diversity of the gut microbiome in dogs with CKD.

There are some limitations to this study, which comprise a small sample size of CKD dogs, particularly those with advanced-stage disease. In this study, the enrollment of advanced-stage CKD dogs fulfills with the strict exclusion criteria which forbid antibiotic or probiotic therapy within 4 weeks was difficult as CKD patients are often prescribed probiotics at early stages of disease from local animal hospitals. Another limitation of this study is the uncontrolled diet and environment of the client-owned dogs. To minimize the limitations in the study, we restricted the dogs involved in the experiment from consuming table food and only provided them with prescription or commercial diets. Additionally, the use of systemic antibiotics and probiotic supplements, which could have an impact on the composition and diversity of gut microbiota, was also restricted.

## 6. Conclusion

Our study demonstrates that the gut microbiome composition in dogs with CKD undergoes a transition from symbiosis to dysbiosis, which can be explained by increased abundance of proteolytic bacteria and decreased abundance of saccharolytic bacteria. Additionally, a few bacterial species known to be associated with systemic inflammatory response in human CKD patients were found to be overgrown in dogs in this study, indicating that a renal–gut axis like that in humans may also exist in dogs. These findings aid in enhancing comprehension of the relationship between CKD and the intestinal microbiome in dogs. Further studies are needed to evaluate the correlation between the gut microbiome composition and microbiome-derived metabolites in dogs with CKD to understand the mechanisms of gut–kidney interactions.

## Data availability statement

The datasets presented in this study can be found in online repositories. The names of the repository/repositories and accession number(s) can be found at: https://www.ncbi.nlm.nih.gov/, PRJNA944392.

## Ethics statement

The animal studies were approved by Konkuk University Institutional Animal Care and Use Committee. The studies were conducted in accordance with the local legislation and institutional requirements. Written informed consent was obtained from the owners for the participation of their animals in this study.

## Author contributions

K-RK: conceptualization, formal analysis, investigation, resource, writing-original draft preparation, writing-review and editing, and visualization. S-MK: formal analysis, data curation, and writing-review and editing. J-HK: conceptualization, writing-original draft preparation, writing-review and editing, supervision, project administration, and funding acquisition. All authors contributed to the article and approved the submitted version.

## Conflict of interest

S-MK was employed by KR Lab Bio Incorporation.

The remaining authors declare that the research was conducted in the absence of any commercial or financial relationships that could be construed as a potential conflict of interest.

## Publisher’s note

All claims expressed in this article are solely those of the authors and do not necessarily represent those of their affiliated organizations, or those of the publisher, the editors and the reviewers. Any product that may be evaluated in this article, or claim that may be made by its manufacturer, is not guaranteed or endorsed by the publisher.
